# Geographic and socioeconomic disparities contribute to variation in opioid prescription patterns in the Medicare Part D population: A cross-sectional study

**DOI:** 10.1016/j.jdin.2025.04.002

**Published:** 2025-04-17

**Authors:** Lauren N. McGrath, Nathan Schedler, Sarah A. Martin, Steven R. Feldman

**Affiliations:** a Center for Dermatology Research, Department of Dermatology, Wake Forest School of Medicine, Winston-Salem, North Carolina; b Department of Dermatology, Wake Forest University School of Medicine, Winston-Salem, North Carolina; c Department of Pathology, Wake Forest School of Medicine, Winston-Salem, North Carolina; d Department of Social Sciences & Health Policy, Wake Forest School of Medicine, Winston-Salem, North Carolina

**Keywords:** dermatology, geographic variation, Medicare Part D, opioid prescription, socioeconomic factors

*To the Editor:* The opioid epidemic poses a significant health risk in the United States and has contributed to the decline in US life expectancy.^1^^,^^2^ Dermatologists prescribe opioids for painful conditions such as hidradenitis suppurativa, postsurgical pain management, and ulcerative conditions like calciphylaxis. However, the geographic distribution of opioid prescriptions and the days of opioid drug coverage per dermatology patient are not well characterized. We assessed the geographic distribution of dermatological opioid prescribing in the United States by analyzing regional, state-level, and city-level prescribing data.

We conducted a cross-sectional study using data on dermatologists who prescribed hydrocodone-acetaminophen, oxycodone-acetaminophen, oxycodone HCl, and tramadol in 2022 from the Medicare Part D Prescribers by Provider and Drug dataset.^3^ Regional, state-level, and city-level analysis included days of opioid coverage per patient, population density, median household income, and percent of residents aged 65+ years living below the poverty line per state. Assumptions were made for omitted data values.

One thousand nine dermatologists prescribed 175,421 days of opioid coverage to 46,855 patients. The mean duration of opioid coverage per patient was 3.74 days. The southern United States had the highest opioid coverage rates, particularly in Alabama, Arizona, and Georgia. The Northeast reported the lowest rates (Table I). North Miami Beach, FL had the highest average days of coverage per patient (131.9 days), totaling over 35 times the national average (3.74 days). High Point, NC and Saint Paul, MN prescribe the least mean days of opioid drug coverage per patient (1.2) (Table II). Cities with higher prescription rates did not show significant differences in population density, income, or poverty levels compared to cities with lower rates (*t*-test; *P* > .05).

**Table I:** Days of opioid coverage prescribed by dermatologists per Medicare Part D patient by US state

State	Total population	Square miles	People per square mile	Median household income	Percent persons 65+ below poverty line	Days of drug coverage per 1000 patients
Alabama	5,074,296	50,650.8	100.2	$59,674	12%	20.03
Arkansas	3,045,637	51,992.8	58.6	$55,432	13%	6.46
Arizona	7,359,197	113,655.3	64.8	$74,568	10%	16.73
California	39,029,344	155,859.3	250.4	$91,551	12%	4.62
Colorado	5,839,926	103,637.1	56.3	$89,302	8%	6.55
Connecticut	3,626,205	4842.4	748.9	$88,429	9%	0.71
Florida	22,244,824	53,653.8	414.6	$69,303	12%	12.06
Georgia	10,912,876	57,717	189.1	$72,837	11%	15.70
Hawaii	1,440,196	6422.5	224.2	$92,458	9%	3.39
Idaho	1,939,033	82,645.1	23.5	$72,785	9%	4.50
Illinois	12,582,032	55,513.2	226.6	$76,708	10%	2.89
Indiana	6,833,037	35,825.9	190.7	$66,785	10%	3.62
Kansas	2,937,150	81,785.5	35.9	$68,925	9%	3.25
Kentucky	4,512,310	39,485.3	114.3	$59,341	13%	9.78
Louisiana	4,590,241	43,212.9	106.2	$55,416	15%	8.11
Massachusetts	6,981,974	7800.9	895	$94,488	11%	2.00
Maryland	6,164,660	9711.2	634.8	$94,991	10%	7.87
Maine	1,385,340	30,845	44.9	$69,543	9%	0.07
Michigan	10,034,118	56,610	177.3	$66,986	10%	2.10
Minnesota	5,717,184	79,631.6	71.8	$82,338	9%	0.97
Missouri	6,177,957	68,746.4	89.9	$64,811	11%	6.03
Mississippi	2,940,057	46,924.5	62.7	$52,719	15%	4.06
Montana	1,122,867	145,550.2	7.7	$67,631	13%	0.56
North Carolina	10,698,973	48,624	220	$67,481	11%	3.90
North Dakota	779,261	68,994.3	11.3	$71,970	10%	1.36
Nebraska	1,967,923	76,817	25.6	$69,597	9%	0.76
New Hampshire	1,395,231	8953.8	155.8	$89,992	8%	0.43
New Jersey	9,261,699	7354.9	1259.3	$96,346	10%	1.56
New Mexico	2,113,344	121,312.8	17.4	$59,726	13%	2.43
Nevada	3,177,772	109,860.5	28.9	$72,333	11%	2.96
New York	19,677,152	47,123.4	417.6	$79,557	13%	0.80
Ohio	11,756,058	40,858.7	287.7	$65,720	10%	1.86
Oklahoma	4,019,800	68,596.7	58.6	$59,673	11%	2.52
Oregon	4,240,137	95,996.7	44.2	$75,657	10%	2.46
Pennsylvania	12,972,008	44,742.3	289.9	$71,798	10%	0.86
Rhode Island	1,093,734	1033.9	1057.9	$81,854	12%	0.50
South Carolina	5,282,634	30,064.3	175.7	$64,115	10%	3.25
South Dakota	909,824	75,807.9	12	$69,728	11%	1.43
Tennessee	7,051,339	41,232.8	171	$65,254	11%	3.30
Texas	30,029,572	261,269.8	114.9	$72,284	12%	6.03
Utah	3,380,800	82,595.5	40.9	$89,168	7%	5.24
Virginia	8,683,619	39,482.1	219.9	$85,873	9%	0.90
Washington	7,785,786	66,455.5	117.2	$91,306	9%	4.16
Wisconsin	5,892,539	54,167.2	108.8	$70,996	10%	0.66
West Virginia	1,775,156	24,041.2	73.8	$54,329	12%	0.87

**Table II:** Days of opioid coverage per Medicare Part D patient by US city, top and bottom 10 US cities

City	State	City population	Square miles	People per square mile	Median household income	Percent persons 65+ below poverty line	Days drug supply per Medicare Part D patient
Top 10 cities							
North Miami Beach	Florida	43,269	4.8	8932.2	$56,122	15%	131.9
Baltimore	Maryland	569,931	80.9	7040.9	$55,198	22%	116.8
Naples	Florida	19,315	12.3	1570	$135,657	4%	113
Williamstown	Massachusetts	4498	3.4	1323.6	$110,855	19%	102.3
West Long Branch	New Jersey	8547	2.9	2992.7	$114,036	7%	45.6
Royal Oak	Michigan	58,053	11.8	4923	$92,799	9%	36
Warren	Michigan	137,111	34.4	3988.5	$60,285	12%	36
Kentfield	California	7410	3	2448.2	$208,977	4%	32.4
Franklin	Kentucky	10,141	14.7	687.7	$54,784	7%	29.5
Redwood City	California	80,529	19.3	4164.7	$137,512	11%	28.4
Bottom 10 cities							
Springfield	Virginia	31,022	7.8	3954.2	$117,598	5%	1.6
Paradise Valley	Arizona	12,672	15.4	823.9	$221,333	1%	1.5
Colorado Springs	Colorado	486,228	201.8	2409	$78,568	6%	1.4
West Chester	Pennsylvania	19,016	1.8	10,300	$71,875	4%	1.4
Glen Ellyn	Illinois	28,304	6.9	4107	$128,132	5%	1.4
Cookeville	Tennessee	34,967	35.8	977.5	$48,094	13%	1.3
Blackfoot	Idaho	12,349	5.9	2084.6	$57,951	9%	1.3
Westminster	Colorado	114,539	31.6	3626.3	$101,529	6%	1.3
High Point	North Carolina	114,339	56.9	2009.2	$63,930	11%	1.2
Saint Paul	Minnesota	303,160	52	5833.1	$67,725	11%	1.2

Opioid prescriptions from dermatologists continue to decline, with an average of 4.4 days per patient in 2014, compared to the current average of 3.74 days per patient.^4^ Dermatologists in the southern United States continue to prescribe more opioids compared to other regions.^4^ Socioeconomic disparities, combined with regional differences in access to dermatologic care and prescribing practices, may contribute to the difference in mean days of opioid drug coverage in some outlier states and cities in the United States. For example, Baltimore, MD is a high-density urban area with a large elderly population and high poverty rates. It has a higher prescription rate (116.8), potentially due to increased healthcare access needs among vulnerable populations.

This study is limited. It includes only Medicare Part D patients, which represent 70% of the total Medicare population in the United States.^3^ Additionally, we are unable to determine the appropriateness of each opioid prescription. In conclusion, while there were no statistically significant patterns, there are some outlier cities where regional influences may factor into opioid prescription patterns. The geographic availability of dermatologists and the cost of dermatologic care in populations experiencing poverty may affect the ability to access dermatological care for postoperative or chronic skin conditions. These may be influencing factors in the regional, state, and city variances in opioid prescription patterns, frequency, and number of coverage days prescribed.

## Conflicts of interest

Steven Feldman has received research, speaking, and/or consulting support from a variety of companies including Galderma, GSK/Stiefel, Almirall, Leo Pharma, Boehringer Ingelheim, Mylan, Celgene, Pfizer, Valeant, AbbVie, Samsung, Janssen, Lilly, Menlo, Merck, Novartis, Regeneron, Sanofi, Novan, Qurient, National Biological Corporation, Caremark, Advance Medical, Sun Pharma, Suncare Research, Informa, UpToDate, and National Psoriasis Foundation. He is the founder and majority owner of www.DrScore.com and founder and part owner of Causa Research, a company dedicated to enhancing patients’ adherence to treatment. The other authors have no conflicts of interest to declare.
